# On Mental and Physical Life in Relation to Time

**Published:** 1863-04

**Authors:** 


### Art. III.—ON MENTAL AND PHYSICAL LIFE IN RELATION TO TIME.

In taking a general view of nature, as comprehending the universe of mind and matter, we are at once struck with the beautiful harmony and order that pervades in perfect continuity its every system. This perfect regularity and surpassing order, as diffused throughout nature, was noticed in very early ages of the world's history by those who had no very precise ideas as to its nature, or the means by which it was produced and maintained. The Pythagoreans, alive to the mysterious beauty of the visible world, called it "Cosmos," to denote at once its order and beauty, while we have the Latin word *mundus* to express the sense of its surpassing loveliness. The true source of this order in nature has been a problem to speculating minds ever since philosophy had its birth. By some it has been referred to the power and varied transformation of certain elements, as air, water, fire, &c.; and by others, again, it was ascribed to the power of numbers. Amongst the Greeks a very special interest gathered round certain numbers, as seven and ten, and certain figures, as the circle and triangle, which came in consequence to be regarded as sacred and perfect. Peculiar feelings became associated with certain periodical seasons, such as the evolutions of the moon, the signs of the zodiac and other cycles, which seemed to the ancients to have a deep significancy in the economy of nature. "The Platonists of the Alexandrian school literally revelled among numbers and forms till they lost themselves among their intricacies and windings. The Platonizing Jew who wrote the Book of Wisdom, caught for a moment a very clear glimpse of the full truth, when he speaks of God having arranged all things in *measure, number,* and *weight.*" It is evident there is still the same earnest desire to find out the means by which unity and order are given to the great Cosmos. All things are governed by laws, and it is a maxim that the end of all science is the discovery of laws. It is the special office of each science to find out what the nature of the laws is in its own sphere. The sum, if we may so speak, of these laws is just the harmony or order established in nature by the Creator. The human mind, the more readily to understand this ultimate law of existence, measures it out into the categories of quantity, quality, &c.; and thus it is that any law that is correctly ascertained originates from one or other of these categories. *Time* is one of the most evident of these categorical elements, for the mind, in looking upon the external world, perceives it always as in space and *time,* which perhaps have no objective reality, but are mere forms of sense or modes of consciousness. This purely metaphysical view of time may prove unphilosophical in certain aspects, but for the object intended it will serve our end as well as that one of Lord Brougham's, which makes time "the succession of ideas, and the consciousness and the recollection we have of that succession." But from Locke we have still a simpler and yet wider definition, which is, the "measure of duration." This inquiry may to many seem fruitless, but to the psychological student it presents one of the most enchanting fields of research. Mental and physical life, in their relations to time, constitute a subject apparently boundless, yet scientifically limited—a subject having many avenues of approach, the grouping of the facts differing according as they are reached through one avenue or another. Indeed, there would seem to be a strong intellectual tendency on the part of mankind to observe the relations of time. In aid of this tendency we have chronology, which fixes on certain great leading events and sets them up as landmarks, around which all other minor incidents are distributed. To these landmarks we are gradually led by experience to associate other events differing in kind—in fact, wholly unconnected except by the law of recurrence.

Thus we measure out the life of man into the landmarks or periods of *infancy*, youth, adult age, and decline, attaching to each epoch a certain series of phenomena. In infancy we look for incessant energy of animal life, the play of instinct, the force of habit, and physical organization in its acme. In youth, we expect to find obedience to instinct replaced by sensation and intelligent desire, the exuberance of organization restrained by the development of mind. In adult man, full development of mind, the will, moral and intellectual, governing his instincts, sensations, and desires; and lastly, in old age, mature calm thought flowing from a passive mind.

Thus we have measured out to us the phenomena of life, each epoch predicating the occurrence of certain events. There are regular epochs, to all appearance, even in the changes on the earth's surface, and in the succession of plants and animals, as disclosed by geological science. There is a beautiful progression, as shown by the science of embryology, in the growth of the young animal in the womb, and the whole life of every living creature is for an allotted period.

Thus all epochs of time, even in their diversity and opposition, conspire to the same end. Incomplete taken in itself, each epoch, joined to that which precedes and to that which follows it, has its share in developing the perfect and beautiful order of nature. We have time, a mighty element, a category binding all the laws of nature together, and bringing them into harmony and order—a generalization, so to speak, of laws, an ultimate fact in the philosophy of existence.

Going from the general to the particular, what are the more striking relations existing between *mind* and this great element, Time?

To know the relations existing between any given elements, it is necessary that we should first know these elements themselves, at least so far as to give us a definite terminology, and enable us to take every step in our argument with security and intelligence. We have settled, for argument's sake, what we mean by the term "*time.*" How do we define the second element, "*mind?*"

"We have, in the instrument of examination, the actual thing to be examined, for we cannot better describe the mental life of man than as embodied in a succession of acts or states of *consciousness*, so continuous as to give and maintain the sense of personal identity."

"Whatever the forms," says Sir H. Holland, "under which we classify and describe the several mental functions, equally must we come to this foundation of mental consciousness, so closely incorporated with intellectual life itself, that every other function may be held to exist only through its manifestations." Here, then, by the analytical process, do we verge all mental acts or functions into a scientific unity—that is, consciousness, which is the medium through which mind acts and reacts on itself, is the representation, or interpreter, of all those actions and reciprocal relations of mental phenomena, as they make up the totality of psychical life. It is with consciousness, then, and in its relation to time, that we have now to deal—with consciousness as an element possessing unity and continuity, and developing itself in the various phases of mental life; and with time as an element almost infinitely divisible, through which our existence is carried forwards in a continuous but ever-changing line.

Taking consciousness as our starting-point, as the ultimate fact of mental life, let us trace out its relations to time. The first question that here suggests itself is one of great importance, and hinges upon what is called the "solidarity," the absolute mutual dependence of the faculties of the mind, the oneness of the mind itself. The point at issue may be shortly stated.

Is this scientific unity, consciousness, limited as to time? In other words, is there any limit to the number of objects co-existing to our consciousness, and are these objects exclusive and single at the same instant of time?

Following the Hamiltonian system of philosophy, which is that of common sense, we find that the first and most general characteristic of consciousness is that it is the recognition by the thinking subject of its own acts or states. The ego distinguishes itself from the non-ego, must know itself before it knows its cognition; thereby denoting a sequence of acts, and of course a limitation as to time. According to the same school, there are certain admitted limitations as to the cognitive power of consciousness, which might serve us as proof of a certain limitation as to time; thus (*a*) consciousness is an *actual* and not a *potential* knowledge. Thus I know, or am able to know, that two and two make four, though that equation be not at the moment the object of my thought; but I am not conscious of this truth unless while *actually present* to my mind. (*b*) Consciousness is an *immediate*, not a *mediate*, knowledge. (*c*) Consciousness supposes a *contrast*, a *discrimination*. I am conscious of a truth only inasmuch as I am conscious of what that truth is—*i.e.*, distinguish it from what it is not; thus the contrasts between the ego and non-ego, subject and object, &c. Here again the sequence and consequent limitation as to time might be argued.

(*d*) Consciousness, again, involves a *judgment*, which is a necessary consequence of the last condition, for I cannot discriminate without at the same time judging.

(*e*) The last and most notable condition of consciousness is *memory*, which also is a corollary of the two last conditions, for without memory my mental acts could not be held fast, compared, distinguished from each other, and referred to self. This notion or recognition of self is possible only through memory, as it arises from the recognised permanence and identity of the thinking subject in contrast to the recognised succession and variety of its modifications. It is memory that links together those infinitesimal moments in the mental succession which otherwise, through their very limitation as to time, would stand isolated and constitute separate existences.

Entering more into detail, we find, on close inspection of our consciousness, three orders of facts, due respectively to *reason*, *sensation*, and the *will*.

(*a*) *Reason* is a mental process by which we compare facts with each other, and mental impressions with external things. Here we find that one faculty or process is necessary above all others to the operation of reason—viz., *memory*. No one can deny that memory is essential to the exercise of thought; that it is the basis of all reasoning. To reason, we must first examine objects or enjoy sensations, but we must remember them before we can compare them, or know their reciprocal bearings. By the property of memory the consciousness of one moment is prolonged into the next, the consciousness of the one moment is recognised as the consciousness of the same being, with that of the next moment; and thus, as we said above, we acquire our notion of self or personal identity. But besides giving continuity to consciousness, memory holds another peculiar relation to time, for it recals the past. "It is memory when the last moment is recalled, when the last idea is recalled." The past is ever being reproduced, and it is owing to this that we have any ideas at all. Memory is necessary to the perception of any idea, and to the discrimination of every relation employed in reason. The mind is one and indivisible, nor can two ideas or thoughts or acts exist in it at one and the same time; it acts always as one being. It recollects, praises, abstracts, judges, imagines; and when you say that it exercises a compound or complex faculty (as the imagination), you only mean that it first exerts one faculty, then another, then a third. "Imagination is not one compound faculty, nor imagining one complex operation of the mind; but the mind in succession *remembers*, abstracts, compares ideas, and reasons or compares judgments; and the whole four successive operations form imagination." (*Brougham.*)

Here, then, an actual sequence, as testified and measured by time, cannot be denied. The simple fact of sequence being admitted, it follows that each mental act must have a definite and exclusive existence in time.

(*b*) *Sensation*—the second fact of our consciousness—may be defined as the consciousness of impression. We know that the mind, strongly intent upon an object, is unconscious of those impressions which are going on around, so that no sensations result from them. And again, all absorbing mental ideas prevent sensations of local impressions unconnected with them; hence wounds are not felt in battle. Why is it, then, that the mind, strongly intent upon an object, is unconscious of existing external impressions? Simply because of the law of limitation as to *time*. Already intent upon or holding one idea, all other impressions are as if they had no existence. The occurrence of some new and strong sensation—as of pain—is well known to be capable of superseding other sensations previously felt, even though the causes of the latter are actually present. This law would seem to explain the action of some of our therapeutic remedies the application of which seems to cause an apparent metastasis of the complaint. Thus, some neuralgic pains are cured by the simple application of a blister to the big toe or any other part equally distant from the part complained of. This can only be explained by supposing that the sensations produced by the blister are greater than the pre-existing pain. Counter-irritation in many other instances would seem to act in much the same manner.

Under this head we have the common faculty of *perception,* which may also be said to be under the same limitation as to time. The mental process that is required to fix the general idea of a landscape upon the memory is, in common language, considered as a simple act of attention. Now, supposing the landscape made up of the usual elements of the beautiful, hill and dale, wood and water, we find it necessary to individualize each of those elements by rapid yet separate acts of attention, before any definite picture can be fixed upon the mind; and even though the laws of vision rendered it possible, it would seem that we cannot by any single effort seize the totality of what is before us. And as the parts are fractionally appropriated by sense, so are they afterwards reproduced by the memory, which can in no other way restore and combine the images deposited for its use. Such a limitation of the mental faculties would seem to argue a singular disproportion between their capacity and the objects to be known; for nothing but multiplicity is ever presented to the observation. But this is only apparent, for by analysis and synthesis in combination, the balance or equilibrium is restored between the powers of knowledge and the objects to be known. "Analysis and synthesis are required to enable the mind to reduce the multitude of objects, the infinitude of Nature, to the limits of its own finite comprehension. We extract the one out of the many, and thus recal multitude to unity, confusion to order. For example, take the perception of a tree. We must first analyse the landscape, so that we may attend to the tree, to the exclusion of the other constituents of the scene. But the tree is not a unity, but a complex union of elements far beyond what our powers can master at once; we must therefore analyse still farther. We accordingly consider *successively* its height, breadth, its shape; we then proceed to its trunk, rise from that to its branches, and follow out its different ramifications. In the same way we fix our attention on the leaves, examine their colour, form, &c. It is only after having thus by analysis detached all these parts, in order to deal with them one by one, that we are able, by reversing the process, fully to comprehend them again in a series of synthetic acts. By synthesis rising from the ultimate analysis, step by step we view the parts in relation to each other, and finally to the whole. We reconstruct them; and it is only through these two counter processes of analysis and synthesis that we are able to convert the confused perception of the tree which we obtained at first sight, into a clear and distinct and comprehensive knowledge." (*Hamilton.*)

Again, if the consciousness be occupied with some visual object, all sounds are lost upon the ear, as if the physical causes did not exist without us. A full orchestra may be executing a chorus of Handel, while the mind is wholly absent from any consciousness of it. As associated with sensation, we may notice the emotions, for, according to Sir W. Hamilton, sensation becomes emotion when controlling intelligence. Experience tells us that a new and strong emotion will often totally obliterate a weaker one existing before. It is equally proverbial, that the most acute feelings—the cause still present—often quit the mind at moments when other objects may have forced themselves upon it, and occurs afterwards with the same intensity as before.

In what way is the *will* related to *time?*

The will, which in its essence is effort, becomes purpose when controlling intelligence, and would seem to come under the same law, as to the exclusiveness of each volitional act at each instant of time.

The intellectual character of any mental process depends on the manner of succession, and especially on the action of the will in determining the result. In how far can the will govern the succession of mental acts or states? In answering this question, let us take the two great faculties of *perception* and *thought.* We can, by the force of will, fix the attention upon *one* voice amidst *many* in a noisy conversation; a still better example is afforded in the simple process of reading, for do we not by the will successively isolate or individualize the letters, words, and sentences, before we can understand the written idea? As an illustration of the power which the mind can exercise by volition over the succession of its thoughts, Sir H. Holland adduces the simple act of *recollection,* "a process by which the mind puts itself into a train of consecutive thought, to obtain hold of some object which is likely to fall into the succession." It is here necessary to notice certain mental processes which are apparent exceptions to this law of volitional limitation. It is well known that a person may perform a difficult piece of *music,* and yet converse freely on some topic quite unconnected with the music. Here we have two psychical processes distinct in themselves, and which to all appearance are absolutely simultaneous in progress and coexisting in time. Some have attempted to explain the above by the law of *habit,* and suppose the person is so well acquainted with his instrument and music that the act becomes an *automatic* one, like the associated movements of muscles in walking, writing, &c. &c.

Feuchterleben, in his *Psychology,* tries to explain these acts by the notion of "*obscure ideas,*" which, according to him, "are ideas such as the mind does not bestow that attention on which is requisite for clear perception, or more properly, sensation with *dormant consciousness.*" This theory of dormant consciousness does not seem very philosophical, for is not sensation the consciousness of impression? and if so, can there be sensation without consciousness? We think not. The knowledge of the existence of pain, for example, is necessary to its existence. Nor does the notion of habit fully explain the anomaly. Is it right to suppose that a purely psychical act can, by habitual repetition or otherwise, become an automatic action, which we understand to mean an instinctive act, without the will? If the music is read, a mental effort (will) is certainly required to individualize the separate bars, as well as to isolate the different musical processes which give effect to the performance. If, on the other hand, the performer knows this music "by heart," *recollection* (will) is certainly necessary to recal and determine the occurrence of the musical ideas. We need not add, that in conversation on any subject the will comes into play in combining ideas, &c. There is no doubt the element time is concerned in the explanation of the above. The consciousness of the performer passes with such rapidity from one subject to another, that we have the effect of simultaneous action, where there is in reality only a succession of states.

The question as to the rapidity with which these acts or states of mind succeed each other is one of great interest and of easy illustration; for proving their limitation in time, we do to a certain extent bear evidence to the rapidity of succession. Nor can we do more than admit a great rapidity of succession, for though physical science, based on the mathematics, can mete out time into determinate divisions which for minuteness are quite inappreciable to the human mind, mental science, from its very nature, can afford no such exact measurement. The rapidity of succession in a quick process of reasoning is so great as even to suggest a unity of time and state. This does not, however, seem to be the case, for in discursive reasoning we individualize, compare, &c., the major and minor terms before we draw our conclusions. The working of the mind in extemporaneous speaking demonstrates also this mysterious rapidity of thought. Perception through the senses also offers a good example of the great rapidity of mental action. In a numerical calculation, the perception of the figures may be so rapid as to give the idea of arriving at the result instinctively. The sense of hearing also is equally quick, for on this depends our comprehension of "human language, the purest earthly revelation of the mind, and harmony, the most refined expression in the sensual world." With what ease does the blind man read from his volume of raised letters, each letter, word, and sentence being the object of tactile perception, before the hidden idea is comprehended! This rapidity is also wonderful in those processes of investigation where two or more of the senses come into *apparent* simultaneous operation. In determining a patient's pulse, the tactile and visual faculties come into action in such rapid succession that they appear synchronous; and it is even upon the belief of this idea that we determine whether the pulsations are 60 or 100 in the minute. Volition, again, works with electrical rapidity. The movement of a limb follows so closely the act of willing, that it is impossible to distinguish the cause from the effect. Of course we refer only to those volitional acts where there is a distinct effort, where consciousness comes into direct action.

It is a well known fact that there are certain conditions which modify the states of consciousness in their relation to time. A great change may take place in the rapidity of succession. During sleep, the imaginative faculties become much more vigorous and uninterrupted, and we have in consequence a long succession of images passing through the mind, with perfect distinctness, in an instant of time, as seen in dreams. In dreams excited by a noise, the same sound may awake the dreamer, and produce a dream which appears to him to occupy a considerable time. Dr. Abercrombie relates the following:—"A gentleman dreamt that he had enlisted as a soldier, joined his regiment, deserted, was apprehended, carried back, tried, condemned to be shot, and at last led out to execution. After all the usual preparations a gun was fired; he awoke with the report, and found that a noise in an adjoining room had both produced the dream and awaked him."

There are many natural agents which seem to have the power of increasing the flow of ideas—*i.e.*, the rapidity of mental succession—as alcohol, opium, henbane, Indian hemp, chloroform, &c. A small quantity of alcohol or opium increases the flow of ideas and the different combinations of thought. A larger quantity prevents our notions of things, and a still larger dose produces loss of intelligence altogether. This is seen also in the action of chloroform. An analogy has been drawn between the states of dreaming and insanity, and in regard to the point at issue the analogy is very close, for in the latter we often find an apparent obliteration of time entirely. The celebrated Robert Hall, when recovering from an attack of acute mania, had very confused notions regarding the flight of time. We give his own words: "You, with the rest of my friends, tell me that I was only seven weeks in confinement, and the date of the year corresponds, so that I am bound to believe you; but they have appeared to me like seven years; my mind was so excited, and my imagination so lively and acute, that more ideas passed through my mind during these seven weeks than in any seven years of my life."

Pathologists know that abnormal states of the brain and spinal cord in many instances influence the rapidity of mental succession. In a case of inflammatory softening of the brain which we saw some time ago, the only disorder of mental function seemed to be great retardation in the succession of his states of consciousness. When a question was asked the patient, he answered very slowly, apparently having great difficulty in combining his ideas and reducing the result of that combination to any kind of formula of speech. After death we found softening of the white matter of the brain. The grey matter was normal, and, as might have been expected, his intelligence was not lost; but, although he understood any question put to him, he was slow in arranging his ideas and conveying them to us in words. Many other instances of disease destroying the relations of the different mental functions might be cited, but it is enough to know that diseases do exist which, by causing structural or functional change in the brain and cord, influence and modify the rapidity of succession of our mental states.

We would draw the following conclusions from the above remarks:

(*a*) There is unity of mind, as expressed in consciousness, which is the point from which we must start in all our investigations into psychical life.

(*b*) There is continuity of consciousness, a prolongation in time, and from this we arrive at our notions of our identity.

(*c*) This unity and continuity of consciousness may constitute the ultimate fact in psychology.

(*d*) A synthesis of this ultimate fact breaks it up into a series of psychical states which are arranged and combined according to certain definite laws.

(*e*) Each mental act or state seems to be exclusive in point of time, no two ideas being under consciousness at one and the same time.

(*f*) These states succeed each other with a rapidity far exceeding all human calculation.

(*g*) Some narcotic drugs, and certain diseased states of the brain, alter and modify the relations existing between our consciousness and the element of time.

(*h*) Consciousness can become self-consciousness only through appreciating the element of time, and the power of doing so is characteristic of the human mind.

